# A comparison of two scales for assessing health professional students’ attitude toward interprofessional learning

**DOI:** 10.3402/meo.v18i0.21885

**Published:** 2013-12-10

**Authors:** Désirée Annabel Lie, Cha Chi Fung, Janet Trial, Kevin Lohenry

**Affiliations:** Department of Family Medicine, Keck School of Medicine of the University of Southern California, University of Southern California, Los Angeles, California, USA

**Keywords:** interprofessional education, health profession students' attitudes, psychometrics, assessment, discriminatory ability, curriculum

## Abstract

**Rationale:**

The validated 19-item Readiness for Interprofessional Learning Scale (RIPLS) is often used for assessing attitudes toward interprofessional education (IPE). The 12-item Interdisciplinary Education Perception Scale (IEPS), also used for this purpose, has not been validated among the professions of medicine, pharmacy, and physician assistants (PAs). The discriminatory ability of the two scales has not been directly compared. Comparison of the two will aid educators in selecting the optimal scale.

**Objective:**

To compare psychometric properties of the RIPLS and IEPS and to examine the ability of each scale to discriminate mean scores among student subgroups (gender, profession, seniority, and prior IPE exposure).

**Method:**

We conducted a cross-sectional (Qualtrics^©^) survey (RIPLS and IEPS) of junior and senior students in medicine (*n*=360), pharmacy (*n*=360), and the PA profession (*n*=106). Descriptive statistics were used to report aggregate mean scores of subgroups. The internal consistency of each scale was assessed using Cronbach's *α*. Concurrent validity was measured by Pearson's correlation coefficients. Independent-sample *t*-tests and analysis of variances (ANOVAs) were performed to assess the discriminatory ability of each scale. Cohen's *d* effect sizes were calculated for all significant pair-wise comparisons.

**Results:**

Response rate was 82%. Cronbach's *α* was 0.85 (RIPLS) and 0.91 (IEPS). The RIPLS discriminated scores by gender among junior students only, and scores by IPE exposure among all students. The IEPS distinguished score differences for the three professions among junior students and by prior IPE exposure for all three professions. Neither scale detected differences in mean scores by profession among all students or by level of training among the three professions.

**Conclusions:**

Neither the RIPLS nor the IEPS has greater discriminatory ability for detecting attitude differences among the student subgroups. Reason for differences may be explained by slightly different scale constructs. The RIPLS is designed to assess students’ own attitude toward interprofessional learning, while the IEPS discerns perceived attitudes about team collaboration for students’ own professions and may be more appropriate for more advanced students.

## Introduction

Interprofessional education (IPE) is commonly defined as ‘any teaching and learning activity that actively promotes collaborative practice’ or ‘occasions when two or more professions learn with, from and about each other to improve collaboration and quality of care’ (1–3). A 2013 Institute of Medicine report (4) updated from the Lancet Commission 2010 report (5) highlights the need for early exposure to IPE and calls for interprofessional training to meet the demands of a changing healthcare system. Important principles for IPE curricula cited in the recently released Interprofessional Education Collaborative (IPEC) Core Competencies for Inter-professional Collaborative Practice report (1) are: being patient-centered, community/population-oriented, relationship-focused, and process-oriented. Given the resurgence and refocus on IPE (6), and as schools, programs, and institutions develop and implement new curricula, reliable, validated tools that are useful across different health professions for tracking the effectiveness of new curricula are needed. Evaluation tools need to discriminate change in knowledge, skills, and attitudes of students in order to assess IPE curricular effectiveness. There is some evidence in the literature to suggest that males versus females (7, 8); those from different health professions (7, 9, 10); and those with disparate prior IPE exposure (7) exhibit varying attitudes toward interprofessional learning. An attitude scale should be capable of distinguishing within these groups and demonstrate longitudinal score changes with IPE curricular exposure. The Readiness for Interprofessional Learning Scale (RIPLS) is a 19-item scale validated for eight health professions (11) that was shown to have reasonable internal consistency and test–retest reliability (12). It contains four subscales (13) of Teamwork and Collaboration, Negative Professional Identity, Positive Professional Identity, and Roles and Responsibilities. The 18-item Interdisciplinary Education Perception Scale (IEPS) was originally developed with a similar intention (14) and validated on 143 students. The IEPS was subsequently remodeled to a 12-item scale with three stable subscales (Competency and Autonomy, Perceived Need for Cooperation, and Perception of Actual Cooperation) to detect changes in learning over time among eight health professions (dietetics, podiatry, physical therapy, occupational therapy, social work, prosthetics, orthotics, nursing, and radiography) in Scotland, United Kingdom.

The IEPS has not yet been validated in the US professions of medicine, pharmacy, and physician assistants (PAs), or among health professions schools in the United States and Canada where training requirements and accreditation standards differ from those in the United Kingdom. Yet concepts and frameworks for IPE have made it to the forefront of these professions via their US national educational organizations (15, 16). In the PA profession, a joint task force from the Society of Teachers of Family Medicine and the PA Education Association made a recent call for interprofessional practice (17, 18). In the pharmacy profession, IPE teaching is required by both the Accreditation Council for Pharmacy Education and the American College of Clinical Pharmacy (19, 20). In medicine, IPE is now an accreditation requirement for US medical schools (1).

Given the recent strong impetus for IPE in the United States and the need for assessing new IPE curricula, we conducted a cross-sectional study to compare the RIPLS and the IEPS for detecting differences in students’ attitudes. Our goal is to provide guidance to educators in the process of selecting a tool for distinguishing IPE attitudes among their students. We hypothesized that both scales can discriminate attitude differences by gender, profession, training level (seniority), and prior IPE exposure. Our secondary aims were to validate the IEPS for assessing the attitudes among these three US health professions and to identify the optimal tool for use among US and Canadian schools and programs.

The institutional review board of the school approved the study status as exempt because anonymous data were collected as a routine part of course administration and teaching.

## Methods

### Setting and participants

This is a one-shot cross-sectional study to explore the psychometric properties of two scales (RIPLS and IEPS). The study was conducted at the Keck School of Medicine and School of Pharmacy at the University of Southern California (USC), where IPE is in early stages of implementation. In 2013, year 1 medical, year 2 pharmacy, and years 1 and 3 PA students participated in a 2-hour small-group IPE session facilitated by faculty pairs from all three professions. The session addressed the IPE competencies of role assumptions and understanding other professions’ roles, and teamwork and collaboration (1, 2). Study participants comprised students from all three professions who were assigned to complete an online survey 4 weeks before the scheduled session. In addition, all senior students from medicine (year 4) and pharmacy (year 3) with minimal prior exposure to IPE were also separately recruited to complete the same online survey during the same time period.

### Survey description and data collection

Our online survey comprised a combination of the 19-item RIPLS (11) and the remodeled 12-item IEPS (20) (see Table 1 for a comparison of items for the two scales). The survey also asked for the student's health profession; gender (M/F); age (less than 25, 25–30, and over 30 years); stage of training (years 1 or 2 categorized as junior and years 3 or 4 categorized as senior students, respectively); prior experience with IPE (none, one, 2–5 occasions, and more than 5 occasions); and prior work experience (narrative response). Prior experience with IPE was categorized as no exposure, slight (one occasion), moderate (2–5 occasions) and high (more than 5 occasions) exposure. The survey was first piloted among 10 faculty members and then revised prior to administration to the students. Results of the survey were shared with teaching faculty who had received a faculty development session before the IPE session, as a trigger for discussion during the IPE session.

**Table 1:** Items for the RIPLS and the IEPS

RIPLS	IEPS
***Teamwork and collaboration*** 1. Learning with other students will help me become a more effective member of a healthcare team. 2. Patients would ultimately benefit if healthcare students worked together to solve patient problems. 3. Shared learning with other healthcare students will increase my ability to understand clinical problems. 4. Learning with healthcare students before qualification would improve relationships after qualification. 5. Communication skills should be learned with other healthcare students. 6. Shared learning will help me to think positively about other professionals. 7. For small-group learning to work, students need to trust and respect each other. 8. Team-working skills are essential for all healthcare students to learn. 9. Shared learning will help me to understand my own limitations. ***Negative professional identity*** 10. I do not want to waste my time learning with other healthcare students. 11. It is not necessary for undergraduate healthcare students to learn together. 12. Clinical problem-solving skills can only be learned with students from my own department. ***Positive professional identity*** 13. Shared learning with other healthcare students will help me to communicate better with patients and other professionals. 14. I would welcome the opportunity to work on small-group projects with other healthcare students. 15. Shared learning will help to clarify the nature of patient problems. 16. Shared learning before qualification will help me become a better team worker. ***Roles and responsibilities*** 17. The function of nurses and therapists is mainly to provide support for doctors. 18. I am not sure what my professional role will be. 19. I have to acquire much more knowledge and skills than other healthcare students.	***Competency and autonomy*** 1. Individuals in my profession are well-trained. 3. Individuals in my profession are very positive about their goals and objectives. 5. Individuals in my profession are very positive about their contributions and accomplishments. 7. Individuals in my profession trust each other's professional judgment. 8. Individuals in my profession are extremely competent. ***Perceived need for cooperation*** 4. Individuals in my profession need to cooperate with other professions. 6. Individuals in my profession must depend upon the work of people in other professions. ***Perception of actual cooperation*** 2. Individuals in my profession are able to work closely with individuals in other professions. 9. Individuals in my profession are willing to share information and resources with other professionals. 10. Individuals in my profession have good relations with people in other professions. 11. Individuals in my profession think highly of other related professions. 12. Individuals in my profession work well with each other.

RIPLS = Readiness for Interprofessional Learning Scale; IEPS = Interdisciplinary Education Perception Scale.

The RIPLS (11) has a score range of 1–5 with higher mean scores representing a more positive attitude toward interprofessional learning, a reported Cronbach's *α* of 0.90 and an intraclass correlation (ICC) of 0.76 (11, 21). The RIPLS was designed for students early in training, and questions ask for students’ opinions about shared learning, team work, and common learning environments for different health professions using a 5-point Likert Scale (from 1 being ‘strongly disagree’ to 5 being ‘strongly agree’). The IEPS uses a 6-point Likert scale with a score range of 1–6 (from ‘strongly disagree’ to ‘strongly agree’). Both scales use summative scores to indicate respondents’ attitude toward IPE with higher scores representing more positive attitudes. Questions are framed around students’ perceptions of their own professions’ capabilities, contributions, collaboration with others, and trust of others’ judgment. The original 18-item IEPS (14) was reduced to 12 items with three subscales (20) and retained a test–retest reliability of 0.6 and a Cronbach's *α* of 0.80. We used the revised 12-item scale for our survey.

We administered the survey using Qualtrics^©^ (Qualtrics Labs, Inc. software, Survey Research Version of the Qualtrics Research Suite, Provo, Utah) 4 weeks prior to the scheduled IPE session, simultaneously to all three health professions’ classes for year 1 medical, years 1 and 3 PA, and year 2 pharmacy students. Students were instructed that

In preparation for the upcoming Interprofessional Education session involving pharmacy, medical and physician assistant students, we would like you to complete this 5 to 10 minute survey so that we may share the results as a starting point for learning together about one another's roles. The survey assesses attitudes about interprofessional learning. There are no right or wrong answers, just your opinion, and your responses are anonymous.

The published and accepted definition of IPE given in this article (1, 2) was provided to students at the beginning of the survey. The same survey was also administered to year 4 medical and year 3 pharmacy (i.e., senior) students in the same time period, with the instruction

We are developing new interprofessional curriculum and would appreciate your completion of this 10 minute survey as a needs assessment for the new curriculum. The survey assesses attitudes about interprofessional learning. There are no right or wrong answers, just your opinion, and your responses are anonymous.

### Data analysis

We used descriptive statistics on both scales to report data in the aggregate, to provide an overview of the performance of each scale. The internal consistency of each scale was assessed using Cronbach's *α*. Both scales (IEPS and RIPLS) use a Likert scale. On the individual item level, the scales appear ordinal but when the items are summed to generate a scale score, the scale becomes interval (22, 23). This procedure is analogous to summing across correct answers on a multiple-choice examination, which makes the scale more interval than ordinal. Norman (24), in a position paper, had shown that ‘parametric methods examining differences between means, for sample sizes greater than 5 (which is the case in our study), do not require the assumption of normality, and will yield nearly correct answers …’. In order to retain the ‘robustness’ in our analyses, we decided to use a parametric approach. We examined the concurrent and discriminant validities of the scales using Pearson's correlation coefficients and comparison of means through independent-sample *t*-tests (gender differences) and analyses of variance (ANOVA) (of score differences). Specifically, we explored the discriminatory ability of each of the two scales for detecting attitude differences by gender, profession, student seniority, and self-reported IPE exposure. Cohen's *d* effect size was calculated to examine the magnitude of the differences found (Formula 1). Formula 1 is defined as:

Cohen's *d* effect size formula using pooled standard deviation.

$$d=M_{1}-M_{2}/\vartheta_{\text{pooled}}\text{.}$$

## Results

### Survey response and participant characteristics

The online survey was administered at baseline with two subsequent weekly reminders to non-respondents. The overall response rate out of a maximum of 826 students (360 medicine, 360 pharmacy, and 106 PA students) for all three professions was 55% in week 1, 68% in week 2, and 94% in week 3. Among the 826 students, there were a total of 675 respondents for an overall response rate of 82%. The response rate was 91% (325/360) among medicine, 70% (250/360) among pharmacy, and 94% (98/106) among PA students for all years of training. The lowest response rate was seen among senior or year 3 pharmacy (41%, 74/181) students. Demographics of students from three professions by age, gender, and seniority are represented in Tables 2 and 3. For all three professions, 93% (627/675) of all students were younger than 30 years, and 60% (404/675) were female. The highest proportion of females was seen in the PA profession (79%, 77/98), followed by the pharmacy profession (68%, 171/252).

**Table 2:** Student demographics (gender, age, seniority, exposure), Keck School of Medicine and School of Pharmacy, 2013

	Professions			
		
	Medicine (*N*=325)	Pharmacy (*N*=252)	Physician assistant (*N*=98)	Total
*Gender*				
Male	170	80	21	271
	52.3%	31.7%	21.4%	40.1%
Female	155	172	77	404
	47.7%	68.3%	78.6%	59.9%
*Age group*				
<25 years	117	142	14	273
	36.0%	56.3%	14.3%	40.4%
25–30 years	187	95	72	354
	57.5%	37.7%	73.5%	52.4%
>30 years	21	15	12	48
	6.5%	6.0%	12.2%	7.1%
*Levels of training within profession*				
First year	172	NA	51	223
	52.9%		52.0%	33.0%
Second year	NA	177	NA	177
		70.2%		26.2%
Third year	NA	75	47	156
		29.8%	48.0%	23.1%
Fourth year	153	NA	NA	119
	48.1%			17.6%
*Exposure to IPE in the past 3 years*				
No exposure	85	101	23	209
	26.2%	40.1%	23.5%	31.0%
Slight exposure	44	45	17	106
	13.5%	17.9%	17.3%	15.7%
Moderate exposure	130	77	34	241
	40.0%	30.6%	34.7%	35.7%
High exposure	66	29	24	119
	20.3%	11.5%	24.5%	17.6%

No exposure = 0 occasions; slight exposure = 1 occasion; moderate exposure = 2–5 occasions; high exposure = more than 5 occasions; IPE = interprofessional education.

**Table 3:** Ethnicity distribution for medicine, pharmacy, and physician assistant students, Keck School of Medicine and School of Pharmacy, 2013

	Medicine (%)	Pharmacy (%)	Physician Assistant (%)
*Junior students*			
Asian	27	67	29
Hispanic	16	3	12
Black/African American	4	4	5
White	51	23	43
Others or unidentified	0	3	11
*Senior students*			
Asian	26	66	24
Hispanic	14	3	14
Black/African American	6	3	5
White	40	26	40
Others or unidentified	0	2	17

### Reliability and concurrent validity of the IEPS and the RIPLS

The Cronbach's *α* for the 19-item RIPLS and 12-item IEPS were 0.85 and 0.91, respectively. Both scales have high internal consistency with the IEPS slightly higher than the RIPLS. The two scales showed moderate correlation as measured by Pearson's correlation coefficient (*r*=0.33). This level of correlation demonstrates that there is enough strength in the relationship of the two scales to infer an overlap in the underlying construct they purport to measure.

### Discriminatory ability of the RIPLS and the IEPS

For the purpose of this study, we specifically explored the discriminatory ability of each of the two scales to detect attitude differences where they are expected (by gender, professions, training levels, and IPE exposures), based on the existing literature (7–10).

## Attitude differences by gender

Wilhelmsson et al. (8) found that regardless of professions, female students were more positive toward teamwork in an interprofessional setting than male students. Since numerous studies have found students’ attitude toward IPE actually become more negative as they progress through training, it is logical to assume that gender differences would be mediated by training level (junior vs. senior students). We ran independent-sample *t*-test to examine whether the IEPS or the RIPLS can detect differences between male and female students across professions at the junior and senior levels separately. When comparing amongst junior (years 1 or 2 of training) students across all three professions, only the RIPLS detected differences in mean scores between male and female students (*t*=2.11, df = 398, *p*<0.05). Female students (*m*=77.7, SD = 8.74) scored significantly higher on the RIPLS compared to male students (*m*=75.9, SD = 8.49). Using Formula 1 to calculate the Cohen's *d* effect size, a small effect between female and male students (*d*=0.28) was found, Neither the RIPLS nor the IEPS detected gender differences by score among senior (years 3 or 4 of training) learners (Table 4).

**Table 4:** Mean scores and discriminatory ability of the RIPS by student gender, Keck School of Medicine and School of Pharmacy, 2013

	*N*	Mean	SD	*t*	Cohen's *d*
*Junior*					
RIPLS^1^					
Female	247	77.7	8.74	*t*=2.11, df = 398, *p*=0.04	0.28
Male	153	75.9	8.49		
IEPS					
Female	247	62.3	6.89	*t*=0.828, df = 398, *p*=0.41	NA
Male	153	61.7	6.82		
*Senior*					
RIPLS					
Female	154	76.3	8.36	*t*=0.766, df = 270, *p*=0.44	NA
Male	118	75.5	9.17		
IEPS					
Female	154	61.1	6.17	*t*= − 0.166, df = 270, *p*=0.87	NA
Male	118	61.2	7.60		

1 Statistically significant at 0.05 level.

RIPLS, Readiness for Interprofessional Learning Scale; IEPS, Interdisciplinary Education Perception Scale; Junior, year 1 medical students; year 2 Pharmacy students; year 1 physician assistant students; Senior, Years 3 and 4 medical students; year 3 Pharmacy students; year 3 physician assistant students; NA, not applicable.

## Attitude differences by professions

Both the RIPLS and IEPS detected score differences *between* the three professions among junior students (RIPLS: *F*_(2,__397)_=10.306, *p*=0.000; IEPS: *F*_(2,__397)_=18.613, *p*=0.000) (Table 5). On both scales, the PA students (RIPLS: *m*=81.37, SD = 6.29; IEPS: *m*=67.10, SD = 5.14) scored significantly higher than medicine (RIPLS: *m*=75.33, SD = 8.92; IEPS: *m*=61.93, SD = 6.24) and pharmacy students (RIPLS: *m*=77.42, SD = 8.59; IEPS: *m*=60.73, SD = 7.22). There were no significant score differences found between medicine and pharmacy students on either scale. Among senior students, only the IEPS detected mean score differences among the three professions (*F*_(2,__272)_=15.251, *p*<0.001). Students in the PA profession had the highest mean score (*M*=61.14, SD= 5.59), followed by medicine (*M*=60.90, SD = 6.54) and pharmacy (*M*=58.89, SD= 6.87). Thus, the IEPS but not the RIPLS detected differences in attitudes between the PA and the other two professions at both the junior and senior levels. All significant pair-wise comparisons resulted in moderate to strong effect sizes as measured by Cohen's *d* that ranged between 0.51 and 0.83 (Table 5).

**Table 5:** Mean scores and discriminatory ability of the IEPS by profession, Keck School of Medicine and School of Pharmacy, 2013

	*N*	Mean	SD	*F*	Cohen's *d*
*Junior students*					
RIPLS^1^					
Med	172	75.33	8.92	*F*_*(2, 397)*_ *=10.306, p < 0.001 PA significantly higher than med and pharm*	NA
Pharm	177	77.42	8.59		
PA^2^	51	81.37	6.29		*PA vs. Pharm: d = 0.53* *PA vs. Med: d = 0.78*
IEPS^1^					
Med	172	61.93	6.24	*F*_*(2, 397)*_ *=18.613, p < 0.001 PA significantly higher than med and pharm*	NA
Pharm	177	60.73	7.22		
PA^2^	51	67.10	5.14		*PA vs. Pharm: d = 0.83* *PA vs. Med: d = 0.71*
*Senior students*					
RIPLS					
Med	150	75.02	9.39	*F*_*(2, 269)*_ *=2.136, p* = 0.12	NA
Pharm	75	77.52	8.00		
PA	47	76.34	7.22		
IEPS^1^					
Med	153	60.90	6.54	*F*_*(2, 269)*_ *=15.183, p* < 0.001 *PA significantly higher than med and pharm*	NA
Pharm	75	58.89	6.87		
PA^4^	47	61.14	5.59		*PA vs. Pharm: d = 0.62* *PA vs. Med: d = 0.51*

1 Statistically significant at 0.01 level.

2 Significant differences found between PA and Medicine; PA and pharmacy only.

RIPLS = Readiness for Interprofessional Learning Scale; IEPS = Interdisciplinary Education Perception Scale.

Junior = medicine year 1, pharmacy year 2, and physician assistant year 1 students; Senior = medicine years 3 and 4, pharmacy year 3, and physician assistant year 3 students; Med = medical students; pharm = pharmacy students; PA = physician assistant students; NA = not applicable.

## Attitude differences by training level

The RIPLS detected mean score differences by student training level (junior vs. senior, i.e., year 1 vs. year 3) amongst PA students (*t*=3.686, df = 96, *p*<0.001) but not among medicine (year 1 vs. year 4) or pharmacy (year 2 vs. year 3) students (Table 6). Year 3 PA students (*m*=76.34, SD = 7.22) had lower RIPLS scores suggesting less positive attitudes compared with year 1 PA students (*m*=81.37, SD = 6.29). The effect size was moderate as measured by Cohen's *d* (*d*=0.74). The IEPS did not detect score differences by student training level (i.e., junior vs. senior students) in any of the three professions.

**Table 6:** Mean scores and discriminatory ability of the RIPLS by seniority (physician assistant students), Keck School of Medicine and School of Pharmacy, 2013

	*N*	Mean	SD	*t*	Cohen's *d*
*Medicine*					
RIPLS					
Year 1	172	75.33	8.92	*t*=0.305, df = 320, *p*=0.76	NA
Year 3 and 4	153	75.02	9.39		
IEPS					
Year 1	172	61.93	6.24	*t*=1.444, df = 320, *p*=0.15	NA
Year 3 and 4	153	60.90	6.54		
*Pharmacy*					
RIPLS					
Year 1	177	77.42	8.59	*t*= −0.088, df = 250, *p*=0.93	NA
Year 3	75	77.52	8.00		
IEPS					
Year 1	177	60.73	7.22	*t*=1.878, df = 250, *p*=0.06	NA
Year 3	75	58.89	6.87		
*Physician assistant*					
RIPLS^1^					
Year 1	51	81.37	6.29	*t*=3.686, df = 96, *p*<0.001	0.74
Year 3	47	76.34	7.22		
IEPS					
Year 1	51	67.10	5.14	*t*=1.485, df = 96, *p*=0.14	NA
Year 3	47	65.49	5.59		

1 Statistically significant at 0.01 level.

RIPLS = Readiness for Interprofessional Learning Scale; IEPS = Interdisciplinary Education Perception Scale; df = degrees of freedom; NA = not applicable.

## Attitude differences by self-reported IPE exposure

Both the RIPLS and IEPS detected score differences by self-reported IPE exposure regardless of profession, gender, and student training level (RIPLS: *F*_(3,__668)_=7.969, *p*<0.001; IEPS: *F*_(3,__668)_=6.321, *p*<0.001) (Table 7). On the RIPLS, students who reported no IPE exposure (*m*=74.36, SD = 8.98) scored significantly lower (i.e., less positive attitudes) than students who reported slight exposure (*m*=77.13, SD = 8.55), students who reported moderate exposure (*m*=77.16, SD = 8.84), and students who reported high exposure (*m*=78.55, SD = 7.21). For the IEPS, students reporting no prior exposure (*m*=60.26, SD = 7.78) scored significantly lower (i.e., less positive attitudes) than those reporting moderate exposure (*m*=62.08, SD = 6.12) and those reporting high exposure (*m*=63.53, SD = 6.35). Similar to the RIPLS, the IEPS did not detect differences in attitude among students reporting slight, moderate, and high exposure. Thus, only attitude differences between the category of ‘no exposure’ and the other three categories (slight, moderate, and high) of IPE exposure were distinguished by both the RIPLS and IEPS. All significant pair-wise comparisons resulted in small to moderate effect sizes as measured by Cohen's *d* that range between 0.26 and 0.55 (Table 7).

**Table 7:** Mean scores for IEPS and RIPLS by prior exposure (all students), Keck School of Medicine and School of Pharmacy, 2013

IPE training exposure	*N*	Mean	SD	*F*	Cohen's *d*
*RIPLS* 1					
No exposure^2^	209	74.36	8.98	*F*_(3, 668)_=7.969, *p*<0.001	NA
Slight exposure	106	77.13	8.55		0.32^4^
Moderate exposure	238	77.16	8.87		0.31^4^
High exposure	119	78.85	7.21		0.55^4^
*IEPS* 1					
No exposure^3^	209	60.26	7.78	*F*_(3, 668)_=6.321, *p*<0.001	NA
Slight exposure	106	61.56	6.46		NA
Moderate exposure	241	62.08	6.12		0.26^4^
High exposure	119	63.53	6.35		0.46^4^

1 Statistically significant at 0.01 level.

2 Three pairs of significant differences found: (1) no exposure and slight exposure; (2) no exposure and moderate exposure; (3) no exposure and high exposure.

3 Two pairs of significant differences found: (1) no-exposure and moderate-exposure groups; (2) no-exposure and high-exposure groups.

4 All effect sizes were computed between this current level and the “no exposure” level.

Slight exposure = 1 occasion; moderate exposure = 2–5 occasions = high exposure = more than 5 occasions; NA = not applicable; IPE = interprofessional education.

In summary, the two scales (RIPLS and IEPS) possess varied ability at detecting differences among gender, professions, and student levels. What both scales are proficient in detecting is levels of IPE exposure across professions. The RIPLS is able to detect gender and profession differences among junior learners, while the IEPS is capable of detecting differences between professions regardless of student levels. Only the RIPLS is able to detect student-level differences, and only within the PA profession.

## Discussion and conclusion

We conducted a cross-sectional survey study of junior and senior students from three US health professions at one institution to compare properties of the RIPLS and the IEPS, two commonly used attitude scales, for discriminating differences about interprofessional learning. We met our hypothesis that both scales can discriminate attitudes by score among these health profession students. Our findings replicated some of the previously established construct validity of each scale and verify the reliability of both scales for measuring attitude toward interprofessional learning (11, 12, 20, 25, 26). Both scales were able to distinguish more positive attitudes among students with greater self-reported prior exposure to IPE. The RIPLS distinguished attitudes between males and females for junior students, and by training level among PA students only; while both the RIPLS and IEPS discriminated differences in attitudes among all three professions for junior students.

There is emerging evidence presented in systematic reviews, supporting the benefits of IPE not only for professional learning but also for improving patient care outcomes and future quality practice (27, 28). A shift toward more positive IPE attitudes during training in part reflects openness to learning and collaboration. The assessment of attitudes toward IPE is relevant in identifying the optimal stage to introduce and reinforce IPE to prepare students for future team collaboration and practice. For educators to accurately assess the impact of IPE curricula at different developmental stages, a sensitive scale is needed. So far, it has been unclear from the literature which of the two scales, the RIPLS or the IEPS, is more appropriate for detecting attitude change for different professions, stages of training, and types of curriculum. We are unaware of any study concurrently assessing the RIPLS and IEPS to compare attitudes of students from the three health professions we examined. Attitudes among students training in the United Kingdom, Canada, and the United States may differ because of differing admissions and accreditation requirements. It is unclear whether findings from the United States, United Kingdom, and Canada are comparable. For example, one UK study (7) reported that the attitudes of medicine, pharmacy, occupational, and physical therapy students as assessed by the RIPLS became more negative over time, while nursing and dentistry students did not exhibit such attitude decline. More negative attitudes toward interprofessional learning over time were also reported by an earlier UK study (9), but the findings are not necessarily comparable to other studies because the authors used different scales: the interprofessional questionnaire, the interim interprofessional questionnaire, and the final interprofessional questionnaire. A recent Canadian study of a curriculum placing students in structured hospital IPE settings reported a significant increase in IEPS score over 5 weeks (29) indicating the sensitivity of the IEPS for detecting attitude change even over a brief, educational exposure. Yet another study examining attitudes of three professions using the RIPLS, IEPS, and the Attitudes Toward Healthcare Teams Scale reported increase in scores (more positive attitude) for all three scales after a single case-based session (30). More such studies (30) are needed to examine and compare discriminatory ability of different attitude scales for IPE.

Our study contributes to the literature by comparing psychometric characteristics of two commonly applied scales during a single administration. Similar to the UK studies (7, 9), we confirmed the decline in attitude toward interprofessional learning by seniority, but our finding applied to PA students only and not to pharmacy or medicine students. We also confirmed the Coster (7) and Wilhelmsson (8) finding of more positive IPE attitudes for females versus males but only for junior students. Thus, administering the RIPLS and IEPS concurrently we verified findings from the existing UK and Canadian studies.

Our study met our hypothesis in establishing that both the RIPLS and IEPS were able to detect differences between levels of IPE exposure; however, the differences were only found between no IPE exposure and any IPE exposure. This could be the result of the criteria set for each level of exposure. We defined slight, moderate, and high exposure using narrow ranges. The scales might have been able to detect differences between exposure levels if the range within each level was greater. A follow-up study is currently in process to establish the ability of either scale to detect the impact of curricular changes (IPE interventions). Other studies have reported that the RIPLS may not be sufficiently sensitive to detect attitude change (10). Our study failed to establish either the RIPLS or the IEPS as superior for finding attitude differences among students from three health professions. We are thus unable to recommend one over the other for tracking longitudinal curricular impact. These findings need to be replicated using assessment before and after the implementation of new IPE curricula. Differential discriminatory ability between the two scales may be expected from their slightly different constructs (see Table 1). The RIPLS was designed to assess novice students’ own attitude toward interprofessional learning, while the IEPS assesses perceived attitudes about team collaboration for students’ own profession. The IEPS may thus be appropriate for advanced or senior students once they have had greater exposure to members of their own profession.

Our study is strengthened by the high numbers of students, a high response rate of over 80% for all three professions and simultaneous administration to all classes. We had the ability to aggregate data across the three professions to increase the power of the subgroup analysis. The survey was conducted anonymously just before a required IPE session and results had no impact on students’ evaluations by faculty; thus, we believe that students were not biased toward any particular response. However, this is a single-institution study and its generalizability to other settings may be limited. The mean scores for both scales were relatively high in each of the subgroups we analyzed and the score differences were small even when statistically significant. Prior exposure to IPE was self-reported and may not accurately reflect true curricular exposure. Finally, the implication for tracking longitudinal attitude change is uncertain given that we examined only differences for non-modifiable subgroups (gender, profession, training level, and prior exposure).

We suggest that IPE educators may need to use both scales to track curricular impact, with a preference for the RIPLS for junior students and the IEPS for senior students who have greater exposure to their own profession and thus can more accurately express their opinions. Our findings support the concept (30) that no single scale may adequately document attitude change. Measures other than attitude scales may be needed to fully account for attitude change with IPE exposure over time. We advocate that educators deploy multiple strategies including qualitative methods such as focus groups (31), narrative analysis of student or faculty reflections (32), and directly observed team behaviors, to evaluate long-term outcomes of incorporating IPE. Future studies will examine subscales within the IEPS and the RIPLS for discriminatory ability; longitudinal change in IEPS and RIPLS scores with increasing exposure to required IPE for the three professions; and the use of mixed methods to assess students’ attitude change over time.

In conclusion, we affirmed the concurrent validity, discriminatory validity, and reliability of the RIPLS and the IEPS for detecting IPE attitude differences in three US health professions. Our findings suggest that either scale may be used to track curricular impact of IPE and that neither is superior to the other.
